# Genome sequencing of captive white tigers from Bangladesh

**DOI:** 10.1186/s12863-024-01239-5

**Published:** 2024-06-06

**Authors:** Ashutosh Das, Md Shahadat Hossain Suvo, Mishuk Shaha, Mukta Das Gupta

**Affiliations:** 1 Department of Genetics and Animal Breeding, Faculty of Veterinary Medicine, Chattogram Veterinary and Animal Sciences University, Khulshi, Chattogram-4225 Bangladesh; 2 Chattogram Zoo, Akbar Shah, Chattogram-4202 Bangladesh; 3 Department of Microbiology and Veterinary Public Health, Faculty of Veterinary Medicine, Chattogram Veterinary and Animal Sciences University, Khulshi, Chattogram-4225 Bangladesh

**Keywords:** White Bengal tigers, Whole genome sequencing

## Abstract

**Objectives:**

The Bengal tiger *Panthera tigris tigris*, is an emblematic animal for Bangladesh. Despite being the apex predator in the wild, their number is decreasing due to anthropogenic activities such as hunting, urbanization, expansion of agriculture and deforestation. By contrast, captive tigers are flourishing due to practical conservation efforts. Breeding within the small captive population can produce inbreeding depression and genetic bottlenecks, which may limit the success of conservation efforts. Despite past decades of research, a comprehensive database on genetic variation in the captive and wild Bengal tigers in Bangladesh still needs to be included. Therefore, this research aimed to investigate the White Bengal tiger genome to create a resource for future studies to understand variation underlying important functional traits.

**Data description:**

Blood samples from Chattogram Zoo were collected for three white Bengal tigers. Genomic DNA for all collected samples were extracted using a commercial DNA extraction kit. Whole genome sequencing was performed using a DNBseq platform. We generated 77 Gb of whole-genome sequencing (WGS) data for three white Bengal tigers (Average 11X coverage/sample). The data we generated will establish a paradigm for tiger research in Bangladesh by providing a genomic resource for future functional studies on the Bengal white tiger.

## Objective

The critically endangered Bengal tiger, *Panthera tigris tigris*, is a native subspecies of the Indian subcontinent. The Bengal tiger population in India started to drop over a century ago. By 1970, less than 2,000 tigers remained in the wild, similar to the global tiger population reduction. Approximately 2,900 wild tigers remain in Indian reserves, making up over 60% of the total number of wild tigers worldwide [1]. According to estimates from the Bengal Tiger Conservation Activity (BAGH) project, there were 114 tigers in the Bangladesh Sundarbans [2]. Despite several conservation efforts, numerous factors, including habitat loss, deforestation, altered land cover, human disturbance of the forests, poaching, hunting, illegal wildlife trade, climate change, natural disasters and inadequate legal frameworks [3], are contributing to the extinction of the tiger population in the Sundarbans.

By contrast, captive tigers are flourishing. Appropriately maintained captive populations of wild animals have been shown to represent a "genetic reservoir" of their natural counterparts, providing insurance against extinction in the wild and aiding in public education, research, and fundraising. Small, isolated populations that experience inbreeding have minimal genetic variety among their individuals and are very vulnerable to extinction. According to estimates, the Bengal tiger population possesses the most genetic diversity, making it the ideal gene pool reservoir for conservation efforts [4, 5]. Using available genomic resources, a high-quality reference genome for Bengal tigers [6] and other tiger genomic data [5, 7–10], we can conduct a comparative genomic analysis and determine the genetic diversity of Bengal tigers. Therefore, we generated this data to compile more comprehensive genomic information, which will be helpful for future research into the variants causing significant colour phenotypes.

Data description.

Following the ethical rules and procedures of Chattogram Zoo Bangladesh, blood samples were taken from three white tiger cubs of three months’ age, including one female and two male cubs. Blood samples were collected aseptically from the cephalic vein using sterile butterfly needles. Blood sample were placed in Vacutainer tubes containing ethylene diamine tetraacetic acid (EDTA) as the anticoagulant. Total genomic DNA was extracted from blood samples using Monarch Genomic DNA Purification Kit (New England Biolabs, UK) according to the manufacturer's guidelines. Thermo Scientific, USA's NanoDropTM One Microvolume UV–Vis Spectrophotometer was used to evaluate the extracted DNA's quality and purity. All samples shown a decent purity with a 160/280 values ranged from 2.06–2.38. For sequencing and library construction (Short Insert library), purified genomic DNA was transferred to Beijing Genomics Institute (BGI, Hong Kong). The DNBSEQ Short-read library preparation instructions provided by the manufacturer were followed for the development of the sequencing libraries. We used a DNBseq platform to do whole genome sequencing(WGS).

**Fig. 1:**
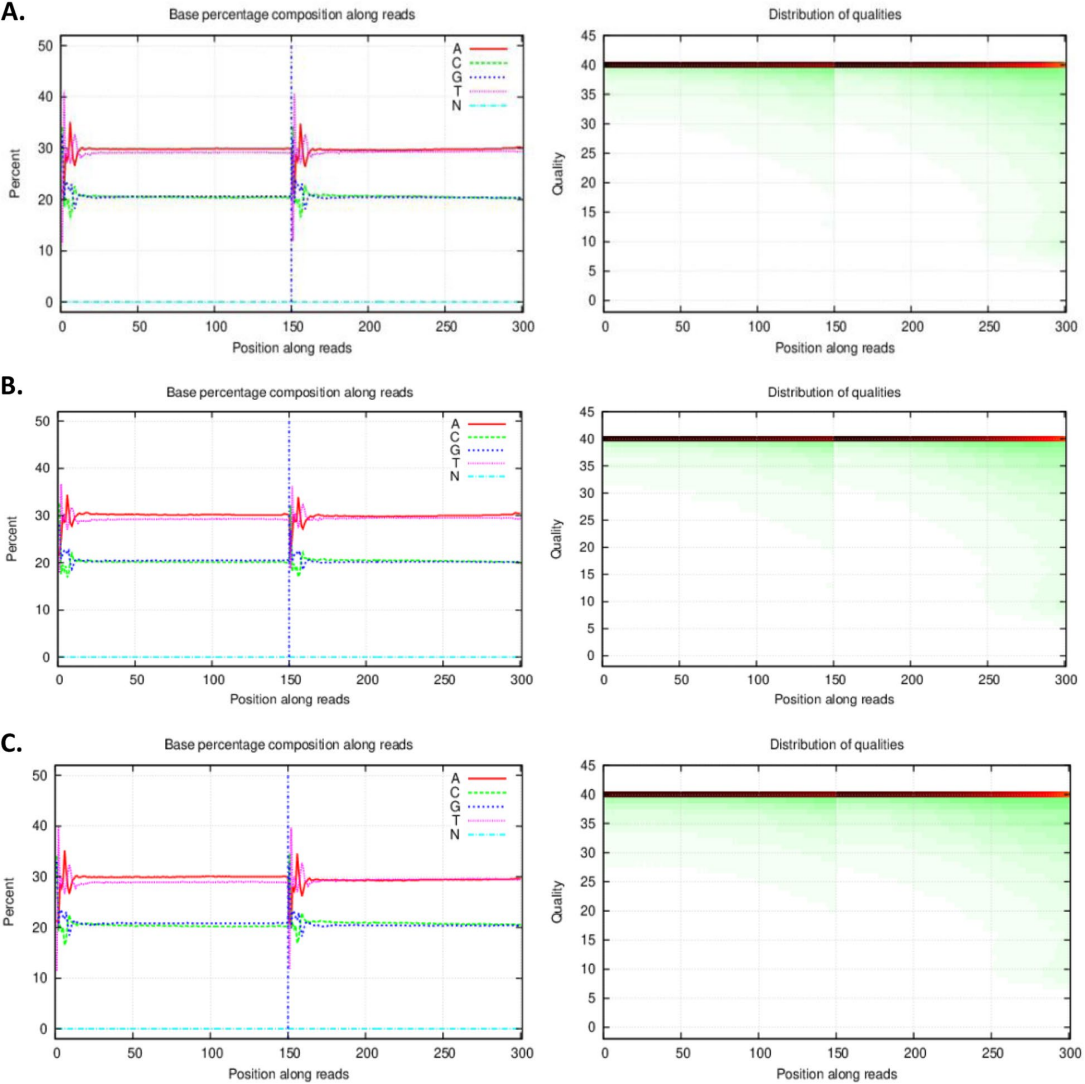
The distribution of base percentage and qualities along reads. In the left figures, x-axis represents base position along reads, y-axis represents base percentage at the position; each color represents a type of nucleotide. Under normal conditions, the sample does not have AT/GC separation. It is normal to see fluctuations in the first several bp positions, which is caused by random primer and the instability of enzyme–substrate binding at the beginning of the sequencing reaction. In the right figures, x-axis represents base position along reads, y-axis represents base quality; each dot represents the base quality of the corresponding position along reads, color intensity reflects the number of nucleotides, a more intense color along a quality value indicates a higher proportion of this quality in the sequencing data. **A**, **B** and **C** represent for sample no. 1, 2 and 3 respectively

High-performance computing resources were used for WGS bioinformatics. Low-quality raw paired readings were removed using SOAPnuke [11] after the raw reads were assessed for quality. In a nutshell, low-quality or adapter sequences in the raw data were filtered first. Many data processing steps were taken to get rid of contaminants and provide reliable data. The filter parameters for the SOAPnuke program were "-n 0.001 -l 10 –adaMR 0.25". The filtering steps were 1) Filter adapter: delete the whole read if the sequencing read matches 25.0% or more of the adapter sequence (a maximum of two base mismatches is permitted); 2) Filter low-quality data: remove the whole sequencing read if bases with a quality value of less than 10 make up at least 50.0% of the read; 3) Eliminate N: Delete the whole read and discard any N information that makes up 0.1% or more of the sequencing read; 4) To obtain clean readings, Phred + 33 was set as the output read quality value for the system. The quality of data was examined after filtering. Base percentage compositions showed all sequenced samples had high-quality data after filtering (Fig. 1) [13]. Burrows-Wheeler Aligner (BWA) software [12] was used to align high-quality reads to the reference *Panthera tigris tigris* genomes, the PanTigT.SI.v4 [6], using the default BWA mem settings.

For three white Bengal tigers that were sequenced, we produced 77 Gb of data (Table 1, Data file 1, 2 and 3) [14–16]. The average genome coverage was 11X. A description of the clean data is shown in Table 2 [17]. Mapping reads encompass 2363074012 base pairs of WGS data which covered 98.46% of the reference tiger genome in the current investigation.

**Table 1:** Overview of data files/data sets

Label	Name of data file/data set	File types (file extension)	Data repository and identifier (DOI or accession number)
Figure 1	The distribution of base percentage and qualities along reads	Document file (.pdf)	Figshare, https://doi.org/10.6084/m9.figshare.24996869 [13]
Data file 1	WGS data of white Bengal tiger sample 1	SRA file (.fastq.gz)	NCBI Sequence Read Archive https://identifiers.org/ncbi/insdc.sra:SRR24305815 [14]
Data file 2	WGS data of white Bengal tiger sample 2	SRA file (.fastq.gz)	NCBI Sequence Read Archive https://identifiers.org/ncbi/insdc.sra:SRR24459545 [15]
Data file 3	WGS data of white Bengal tiger sample 3	SRA file (.fastq.gz)	NCBI Sequence Read Archive https://identifiers.org/ncbi/insdc.sra:SRR24632529 [16]
Table 2	Basic statistics of whole genome sequence data for captive white tiger from Bangladesh	Document file (.docx)	Figshare, https://doi.org/10.6084/m9.figshare.25902517.v1 [17]

**Table 2:** Basic statistics of whole genome sequence data for captive white tiger from Bangladesh

Sample Name	Clean Reads	Clean Base	Read Length	Q20(%)	Q30(%)	GC(%)
WBT_01	147,848,227	44,354,468,100	PE150	96.92	92.55	41.15
WBT_02	145,372,272	43,611,681,600	PE150	97.07	93.01	40.72
WBT_03	88,031,025	26,409,307,500	PE150	97.29	92.55	41.35

*WBT* White Bengal tiger

Limitations.

Since all the samples come from individuals from the same parent, performing a genome-wide association study to identify genomic regions associated with a particular phenotype were not possible.

## Data Availability

Figure 1 and Table 2 described in this Data Note can be freely and openly accessed on FigShare (https://figshare.com/) [13, 17]. The data described in this Data note can be freely and openly accessed from the NCBI Bioproject PRJNA961947. Raw data have been deposited separately in the Sequence Read Archive (SRA, https://www.ncbi.nlm.nih.gov/sra) with open accession ID SRP434520 [14–16].

## Abbreviations

BWA: Burrows-Wheeler Aligner
DNA: Deoxyribonucleic acid
Gb: Gigabase
WGS: Whole genome sequencing
